# Improving case-finding of long-term health problems in disadvantaged communities: A Policy-focused Evidence Brief^⋆^

**DOI:** 10.1016/j.puhip.2026.100769

**Published:** 2026-03-12

**Authors:** Adnaan Ghanchi, Anita Patel, Helena Painter, Isla Kuhn, Anna Gkiouleka, Ian Holdroyd, Danielle Lamb, John Ford

**Affiliations:** a Department of Public Health and Primary Care, University of Cambridge, UK; b Wolfson Institute for Population Health, Queen Mary University of London, UK; c University of Cambridge Medical Library, School of Clinical Medicine, Cambridge, UK; d NIHR Applied Research Collaboration, UCL, UK

**Keywords:** Case finding, Health inequalities, Chronic disease screening

## Abstract

**Current challenges:**

Case finding, which actively identifies undiagnosed conditions, is key to tackling the growing chronic disease burden, especially in disadvantaged groups. Diseases like type 2 diabetes, cardiovascular disease, and chronic obstructive pulmonary disease already cause millions of preventable deaths, with numbers rising due to aging populations, urbanization, and widening inequalities. The poorest communities bear the heaviest load, with higher disease rates and lower diagnosis. Traditional healthcare models, which wait for patients to seek care, leave millions undiagnosed and untreated. Proactive case finding is a proven way to reduce disparities, improve outcomes, and ease the strain on healthcare systems.

**Key evidence to inform policy:**

This review identified two main approaches: targeted searches of primary care electronic health records (EHRs) to identify and engage high-risk individuals, and opportunistic screening in community, workplace, and emergency department settings. Systematic reviews and smaller observational studies, predominantly from high-income healthcare systems, support community-based health checks in trusted venues, workplace hypertension screening, and follow-up of elevated blood pressure readings in emergency departments, though evidence for these approaches is more limited. A stepped-wedge RCT found that targeted outreach nearly doubled diagnosis and treatment rates for cardiovascular risk factors compared with opportunistic case-finding (19.7% vs 10.8%). Meta-analyses of over 480,000 women found that self-sampling approximately doubled cervical screening uptake, with uptake increasing threefold when combined with community health worker support among under-screened ethnic minority and socioeconomically disadvantaged women. A cluster-randomised trial of over 74,000 participants showed that active case-finding using symptom questionnaires and handheld flowmeters identified four times more undiagnosed COPD than opportunistic screening, and was cost-effective at £16,596 per QALY gained.

**Further considerations and implications:**

This review identifies targeted case-finding in underserved communities as an evidence-based, high-impact strategy for early detection of chronic disease, provided it is integrated with sufficient downstream diagnostic and treatment capacity.

## Highlights

• Case-finding strategies, including electronic health record searches, identify undiagnosed conditions in disadvantaged communities.
• Community health checks, workplace screenings, and outreach in trusted venues (churches, pharmacies) improve detection.
• Self-sampling boosts cervical cancer screening participation among those avoiding traditional settings.
• Disease-specific risk tools, including COPD questionnaires and CVD risk profiling, identify at-risk individuals early.

## 1 Current policy challenges

**Fig. 1:**
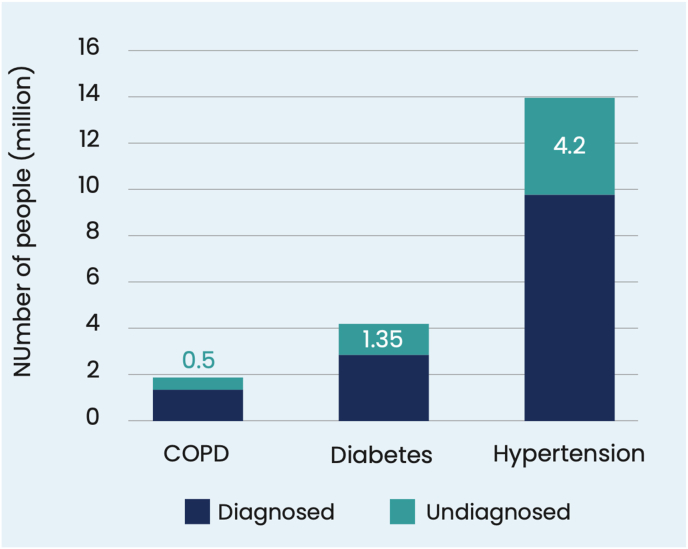
Estimated number of people with diagnosed and undiagnosed COPD, diabetes and hypertension in England.

Sources: COPD – Stone and colleagues Prevalence of Chronic Obstructive Pulmonary Disease in England from 2000 to 2019. Int J Chron Obstruct Pulmon Dis. 2023 [4]. Diabetes – Health Survey for England 2021 [5]. Hypertension – Office for National Statistics 2023 - Risk factors for undiagnosed high blood pressure in England: 2015 to 2019 [6].

Across the world, the poorest in society are the most likely to have trouble accessing healthcare and concurrently the most likely to suffer from multiple chronic diseases. In the UK, the most socioeconomically disadvantaged communities have at least a 50% higher prevalence of cardiovascular disease (CVD), diabetes, chronic pain, substance misuse and anxiety and depression. [1]. Chronic obstructive pulmonary disease (COPD), a smoking related lung disease, is three times more common in the most deprived populations [1]. Some ethnic minority groups also have higher CVD, COPD and diabetes prevalence and complications. For example, South Asian minority groups are at increased risk of CVD and have mortality rates almost 50% higher when compared to non-South Asian populations [[1], [2], [3]] [see Fig. 1].

**Fig. 2:**
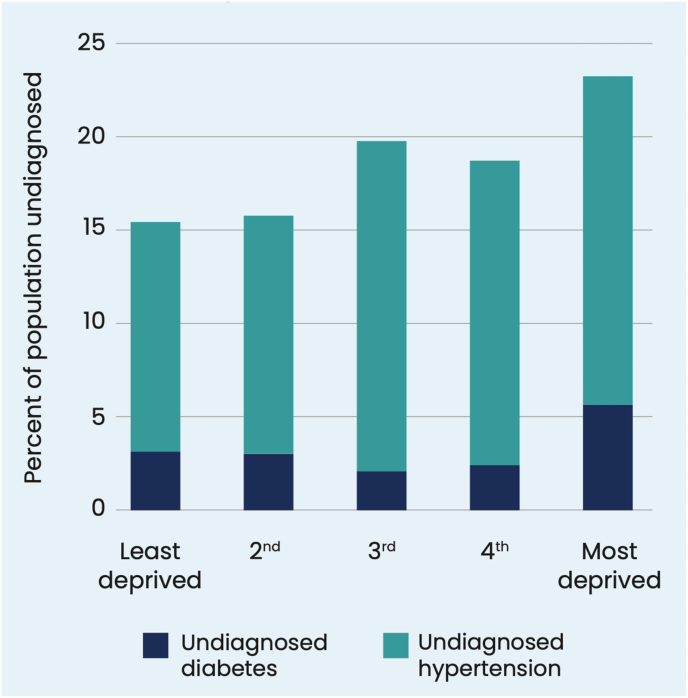
Estimated proportion of people with undiagnosed diabetes and hypertension across socio-economic groups.

Source: Health Survey for England 2021 [5]. Note: No data is available for COPD.

People living in poorer areas are more likely to have an undiagnosed health problem. The Health Survey for England found that there are twice as many people with undiagnosed diabetes in deprived areas compared to affluent areas and 50% more people with undiagnosed hypertension [5, Fig. 2]. There are an estimated 4.2 million people with undiagnosed hypertension, 1.35 million with undiagnosed diabetes and 500,000 with undiagnosed COPD [5,4,6]. Based on data from the Health Survey for England and a study of primary care records, the number with undiagnosed high cholesterol is even higher at 9.9 million [7].

It is estimated that £68bn could be saved, 4.9 million quality adjusted life years (QALYs) gained, and 3.4 million CVD cases prevented over 25 years if those in England with the six CVD high risk conditions (hypertension, diabetes, non-diabetic hyperglycaemia, atrial fibrillation, chronic kidney disease and high cholesterol) were diagnosed and managed at current levels [8].

Given the significant individual and collective costs of undiagnosed long-term conditions, especially in disadvantaged groups, finding a solution is vital. Better identification and management of these risk factors and conditions would improve health and reduce costs. Here we explore the evidence of what works to identify people from disadvantaged backgrounds, specifically lower socio-economic and ethnic minority groups, with undiagnosed health conditions through case finding.

## 2 Approach to collating evidence

We undertook a rapid review of the evidence to identify key policy and practice recommendations. We sought to identify the most relevant and robust literature on the topic to inform decision making, rather than attempting to identify every article covering a wide topic. We identified studies from the Health Equity Evidence Centre Living Evidence Maps of what works to address inequalities, a search of MEDLINE and snowball searching using Litmaps. We prioritised latest reviews and those of greatest relevance to high-income health care systems. Our focus was on interventions which facilitate the identification of undiagnosed long-term conditions in disadvantaged groups. In total we prioritised 22 studies. The evidence reviewed derives predominantly from high-income healthcare systems, particularly the UK, therefore implementation may require adaptation for other contexts. We narratively synthesised these studies focusing on strongest and most relevant evidence.

## 3 Key evidence to inform policy

**Fig. 3:**
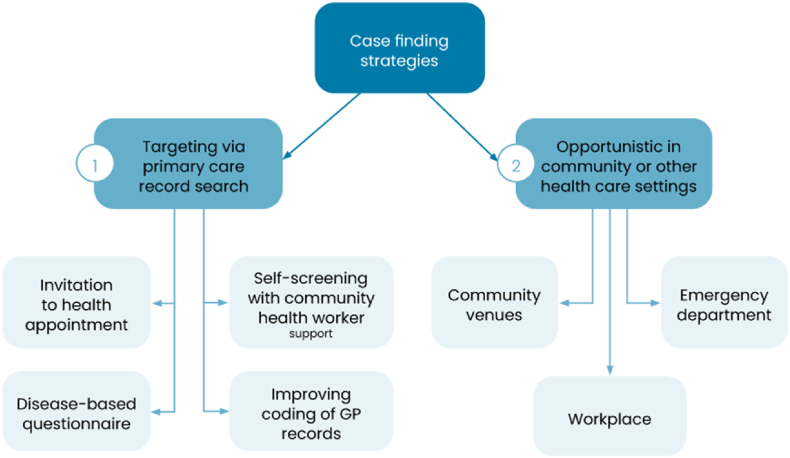
Case finding strategies for disadvantaged groups.

We found two main starting points for case finding of disadvantaged groups – 1) searching the primary care electronic patient record to identify at risk people and engaging with them and 2) opportunistic engagement in the community, workplace or when patients attend other health care settings, such as emergency department [Fig. 3]. We found multiple strategies that support the identification of health problems in disadvantaged groups. It is unlikely that a single programme or intervention will identify the majority of cases, but rather a multicomponent programme that seeks to identify cases through multiple approaches and settings.

### 3.1 Interventions initiated through primary care electronic health record (EHR)

In primary care, targeting patients through searches of the electronic patient record is more effective than opportunistic case finding, such as receptionists or GPs opportunistically asking people when they attend the surgery. Hemming and colleagues (2016) undertook a randomised control trial exploring whether *targeted* case-finding for prevention of CVD is more effective than *opportunistic* case-finding in primary care [9]. A nurse in primary care identified high risk (CVD risk of >20%) and undiagnosed patients between 35 and 74 years from patient records. Patients were invited for assessment (including BP measurement) and referred appropriately to their GP for treatment if required. The study showed an almost doubling of diagnosis with 19.7% of patients being diagnosed and prescribed antihypertensives or statins in the targeted group versus 10.8% of patients enrolled in opportunistic case-finding.

Kypridemos and colleagues undertook a microsimulation study to compare the current model of universal health checks (i.e. everyone 40-74 years without pre-existing health problems) compared to targeting the most deprived areas [10]. The authors found the universal screening would avoid 19,000 CVD events and 3000 deaths. Whereas the targeted approach to the most disadvantaged 40% of the population would avoid 17,000 events and 2000 deaths but for considerably fewer resources.

#### 3.1.1 EHR search followed by disease-based questionnaire

COPD has a high prevalence in socio-economically disadvantaged areas. One Dutch mixed methods observational study compared case finding for COPD in practices in low, moderate and high socioeconomic areas using patient questionnaires. They found more high-risk individuals (older and more current smokers) and more cases of COPD in the low SES practices compared to in moderate or high SES practices [11].

Patient questionnaires and handheld flow meters may be combined with searches of the electronic health record to identify people at risk of COPD. It is particularly suited to COPD because there are currently substantial waiting times for spirometry, the medical investigation required for a diagnosis, and since 90% of people with COPD have previously smoked they can be identified by smoking records [12].

The CAPTURE symptom questionnaire with or without peak flow measurement, a handheld device for helping to diagnose lung problems, is one example that has been shown to be sensitive and specific in identifying COPD that requires treatment [13]. The TargetCOPD score uses a risk score incorporating smoking status, respiratory symptoms and other risk factors using routine primary care data to identify high risk patients [14].

The TargetCOPD trial was a major UK-based cluster-randomised control trial with over 74,000 participants comparing targeted case finding for COPD to usual care [15]. Participants were randomised to receive screening questionnaires opportunistically during visits to the GP or actively where questionnaires and reminders were posted to eligible participants in addition to opportunistic screening. Those who scored highly for respiratory symptoms were offered referral for diagnostic spirometry. Targeted case finding identified statistically significantly more undiagnosed COPD compared to opportunistic case finding (4% vs 1%). A subsequent analysis found that patients diagnosed through COPD case finding were less likely to be added to the COPD register and less likely to be offered support for their condition [16]. Long term data from TargetCOPD found no difference in hospitalisations or mortality between the groups over 4 years [17].

A cost-effectiveness analysis of TargetCOPD found that it was more cost effective than opportunistic case finding [18]. Active case finding for COPD compared to opportunistic case finding had an incremental cost effectiveness ratio (ICER) of £16,596 per quality adjusted life year (QALY). A Canadian budget impact analysis modelled the total medical costs of eight different opportunistic COPD case finding strategies [19]. The researchers found a symptom questionnaire and diagnostic spirometry was most cost-effective with a total budget cost of $423 million. A similar microsimulation economic analysis in China evaluated cost-effectiveness of a population wide case finding programme [20]. They found that case finding with a symptom questionnaire and handheld flow meters offered to the entire population over 35 years old (irrespective of smoking status) was the most cost-effective.

#### 3.1.2 Identification of non-responders of cervical screening followed by self-screening and community health worker support

Self-sampling may help to find additional cases missed by the national cancer screening programme. A meta-analysis looking at the effectiveness of self-sampling on cervical cancer screening uptake identified 154 studies globally (33% low-income countries) including 482,271 women [21]. Many studies were conducted in marginalised, under screened populations, although others were looking at the general population. They found that self-sampling almost doubled the probability of cervical cancer screening uptake when compared with samples collected by clinicians, with an opt out approach being most effective. The effect size was greater among previously unscreened women compared to the general population.

Another meta-analysis of 33 studies (26 RTCs in high-income countries, including two in England) similarly found that self-sampling more than doubled screening uptake in screening non-respondents, with greater increases among lower socioeconomic status women [22]. Offering self-testing combined with education through community outreach by health workers had an additional impact, increasing screening uptake by three times in five RCTs of participants from ethnic minority backgrounds or from medically underserved communities. Ethnic minority women and socioeconomically disadvantaged women had higher rates of screening when they had support from a community health worker supervising their self-sampling. Sending reminders to attend clinician delivered screening was also effective in increasing uptake.

Evidence shows that self-sampling shows similar clinical accuracy in detecting cervical pre-cancer as clinician collected samples and addresses many of the barriers to standard cervical screening such as stigma, travel issues to the clinic, taking time off work and arranging childcare which may be beneficial to those from more deprived backgrounds [23,24].

Sun and colleagues considered the cost-effectiveness of different strategies in increasing uptake of cervical cancer screening in underserved women in a systematic review of 17 European studies [25]. Self-sampling as opposed to clinic-based sampling was found to be a preferred and cost-effective add-on to usual screening invitations for underserved women.

#### 3.1.3 Improved coding of GP records

Some patients may receive treatment for a health problem,but not have a diagnosis on their electronic record and therefore miss out on follow-up, such as annual health checks. A 12 month audit in 2018 across 48 practices in East Berkshire identified an additional 6167 people with hypertension over 12 months through searches for people who had been investigated and received treatment for a condition, but not had a diagnostic code entered into their patient record [26].

### 3.2 Opportunistic case finding in the community, workplace or emergency department

Several studies have examined opportunistic case finding in the community, such as BP checks in pharmacies or mobile units, case finding in workplaces or opportunistic case finding when patients attend the emergency department.

#### 3.2.1 Opportunistic community-based case finding

Fleming and colleagues (2015) reviewed 73 studies reporting the effectiveness of community-based screening including self-screening for hypertension [27]. The highest proportions of eligible participants screened were through mobile units (range 21%–88%) and pharmacies (range 40%–90%). Roberts and colleagues (2016) looked specifically at reaching diverse groups for the NHS Health Check in Buckinghamshire through community venues compared to general practice [28]. Of 3849 community-based health checks, 11% involved users of South Asian ethnicity (compared to 3% in primary care). Mosques and bus stations were the venues with the broadest reach to underserved groups. An ethnographic study of 20 participants in inner-city Bristol, predominantly from the Afro-Caribbean community, showed that having Health Checks endorsed by trusted community members in lay language supported attendance among disadvantaged groups. Participants indicated that it was their respect for and loyalty to the engagement worker (of similar ethnic background to them) which prompted them to attend the Checks and venues which were both familiar and convenient [29].

##### 3.2.1.1 Case finding in the workplace

People in low-paid and manual jobs often face being both at high risk of ill health and finding it difficult to take time off to attend preventative health care appointments. Case finding in workplaces potentially offers the opportunity to target low-paid and manual jobs while reducing barriers to preventative care.

A few small-scale studies have explored case finding in workplaces to reach disadvantaged groups. In 2015, Legorreta and colleagues looked at hypertension screening in asymptomatic employees in US workplaces using insurance claims data [30]. Based on 4414 people with high blood pressure, the authors found that African Americans and those on lower incomes were more likely to be diagnosed in the workplace with hypertension compared to white Americans and those on higher incomes. A further US-based study evaluated hypertension case finding in taxi drivers with events at taxi garages, airport holding lots, and app-based driver centers [31]. Most drivers were male, mean age was 47 and half of drivers were not proficient in English. The scheme identified 297 people with undiagnosed hypertension (14% of total and 30% of high risk).

Researchers at the University of Southampton evaluated a case finding initiative on their campus which involved a health questionnaire and BP check [32]. A total of 653 staff and students took part, and the authors found higher rates of hypertension in male manual workers compared to what would have been expected for the age and sex. Finally, Sonkodi and colleagues evaluated hypertension case finding in a salami factory in Hungary which involved a questionnaire and BP check [33]. In total 1012 factory workers were screened, and of the 260 employees with high blood pressure, 100 (39%) were undiagnosed.

#### 3.2.2 Opportunistic case finding in the emergency department (ED)

Patients from minority ethnic groups and more socio-economically disadvantaged areas are more likely to attend ED and therefore screening for BP with community pharmacy or GP confirmation may be effective for disadvantaged groups [34,35]. Michaud and colleagues (2020) reviewed 10 studies that assessed the use of screening for hypertension (measuring BP and making appropriate referrals for diagnostic confirmation) in the emergency department (ED) [36]. The authors found that 43.4% of patients with elevated blood pressure during the ED visit were subsequently diagnosed with hypertension.

## 4 Further considerations and implications

### 4.1 Limitations

Limitations of this study include heterogeneity of disadvantaged populations. The term “disadvantaged” encompasses a broad range of individuals, including those from lower socioeconomic backgrounds, ethnic minority groups, and people facing language barriers or mistrust of healthcare institutions. While certain case-finding approaches may work well for some groups, they may be less effective for others.

Many studies report success in identifying undiagnosed conditions but do not track long-term health outcomes, cost-effectiveness, or the sustainability of interventions. Without this data, it remains unclear whether the identified strategies lead to lasting health benefits or reductions in healthcare costs over time.

Finally, even when effective case-finding methods are identified, real-world implementation presents its own set of challenges. Practical barriers such as workforce capacity, financial constraints, and the willingness of patients to engage with screening and follow-up care can all affect the success of these strategies.

### 4.2 Implications

Clinicians and patients are generally in favour of case finding. However, case finding inherently increases primary care workload with more patients requiring diagnosis and management [37]. Formal diagnosis usually requires additional testing and any intervention that will increase the number of people with chronic disease needs additional resource. Concerns also remain regarding the potential harm to patients through overdiagnosis [38,39].

Policymakers should consider prioritising EHR-based targeted case-finding, where the evidence is strongest, while supplementing with community and workplace approaches to reach those not engaged with primary care. Integrating these strategies into routine healthcare delivery will require addressing barriers to engagement, including trust, accessibility, and resource allocation. Furthermore, long-term evaluations are needed to assess sustainability, cost-effectiveness, and health outcomes over time. With undiagnosed conditions disproportionately concentrated in the most deprived communities and modelling suggesting substantial cost savings from better detection, the case for scaling up targeted case-finding is both a health equity imperative and an economic one.

## Ethics statement

Not applicable, as this study is based on a literature review.

## Availability of data and materials

The data supporting this article's conclusions are available in the referenced studies.

## Authors' contributions

JF conceptualised the study. AP and IK led the study identification and extraction. AGh, AP, HP, IH, DL and AGk supported the interpretation of the findings. AGh and JF led the manuscript writing. All authors contributed to and approved the final manuscript.

## Disclosure statement

This report is independent research supported by the National Institute for Health and Care Research ARC North Thames and NHS England. The views expressed in this publication are those of the author(s) and not necessarily those of the National Institute for Health and Care Research, NHS England, or the Department of Health and Social Care.

## Funding

This study was commissioned by NHS England.

## Declaration of competing interest

The authors declare the following financial interests/personal relationships which may be considered as potential competing interests:This report is independent research supported by the National Institute for Health and Care Research ARC North Thames and NHS England. The views expressed in this publication are those of the author(s) and not necessarily those of the National Institute for Health and Care Research, NHS England, or the Department of Health and Social Care.
